# New insights into the taxonomy of tribe Euclidieae (Brassicaceae), evidence from nrITS sequence data

**DOI:** 10.3897/phytokeys.100.24756

**Published:** 2018-06-21

**Authors:** Hongliang Chen, Ihsan A. Al-Shehbaz, Jipei Yue, Hang Sun

**Affiliations:** 1 Key Laboratory for Plant Diversity and Biogeography of East Asia, Kunming Institute of Botany, Chinese Academy of Sciences, 132 Lanhei Road, Kunming, Yunnan 650201, China; 2 University of Chinese Academy of Sciences, Beijing 100049, China; 3 Missouri Botanical Garden, P.O. Box 299, St. Louis, Missouri 63166-0299, USA

**Keywords:** Euclidieae, Brassicaceae, Cruciferae, *Solms-laubachia*, Phylogeny, nrITS

## Abstract

As currently delimitated, the species-rich mustard tribe Euclidieae DC. (Brassicaceae) comprises 28 genera and 152 species distributed primarily in Asia. To date, no tribe-wide comprehensive phylogenetic analysis has been conducted. In this study, sequence data from the nuclear ribosomal internal transcribed spacer (nrITS) region of 82 species in all 28 genera of Euclidieae were used to test its monophyly and infer inter- and intra-generic relationships within. Phylogenetic analyses revealed that *Rhammatophyllum* and *Sisymbriopsis* are embedded within *Solms-laubachia* s.l., and *Solms-laubachia lanuginosa* (*Eurycarpus lanuginosus*) fell outside the tribe. Therefore, *Solms-laubachia* s.l. as currently recognized is not monophyletic and its generic delimitation needed further study. Besides, our results suggest that the genera *Lepidostemon*, *Neotorularia*, and *Tetracme* are polyphyletic.

## Introduction

The Brassicaceae (Cruciferae) includes 52 tribes, 341 genera, and 3,997 species (BrassiBase: https://brassibase.cos.uni-heidelberg.de/, accessed 5 February 2018, Koch et al. 2012, Kiefer et al. 2014) distributed worldwide, primarily in the temperate regions (Al-Shehbaz 1984, Al-Shehbaz et al. 2006). The family is academically and economically important (Franzke et al. 2011, Al-Shehbaz 2012, Huang et al. 2016), as it contains the well-known model plant species *Arabidopsis thaliana* (L.) Heynh. and many crops (e.g., cabbage, cauliflower, turnip, rape, canola, radish, wasabi) and ornamentals (e.g., species of *Lobularia* Desv., *Iberis* L., *Hesperis* L., *Matthiola* W. T. Aiton). Although the family is easily recognized morphologically, it is often difficult to assign an individual plant to a given genus, and there are tremendous controversies on generic delimitations and tribal assignments (Al-Shehbaz et al. 2006, Al-Shehbaz 2012). The total number of tribes and genera they include varied among different systems. For example, Schulz (1936) and Janchen (1942) recognized 19 and 15 tribes, respectively. By contrast, others (e.g., Zhou et al. 2001, Appel and Al-Shehbaz 2003) adopted an alphabetical arrangement of genera. The use of molecular sequences to infer phylogenetic relationships during the past two decades have greatly improved our understanding of the evolution within the Brassicaceae. That led Al-Shehbaz et al. (2006) to propose the first phylogenetic tribal classification system based on prior molecular studies, especially the chloroplast *ndh*F sequences (Beilstein et al. 2006), and had since been expanded to include 52 tribes (Al-Shehbaz 2012, German and Friesen 2014, Chen et al. 2016). Generic boundaries had also been redefined, and studies on *Solms-laubachia* s.l. (Yue et al. 2006, 2008), *Eutrema* R. Br. (Al-Shehbaz and Warwick 2005), *Microthlaspi* F. K. Mey. (Ali et al. 2016), to name a few, demonstrate that trend.

The tribe Euclidieae DC. was established ca. 200 years ago (de Candolle 1821), and it has been accepted in subsequent tribal classifications, though the delimitation of its component genera remained controversial. Of the 14 genera recognized by Schulz (1936) in the tribe, only *Euclidium* W. T. Aiton and *Lachnoloma* Bunge were maintained by Al-Shehbaz (2012) (Table 1). As currently delimited (Warwick et al. 2007, Al-Shehbaz and German 2016, BrassiBase) (Table 1), the tribe comprises 28 genera and 152 species, including the species-rich *Solms-laubachia* Muschl. (33 spp.), *Strigosella* Boiss. (24 spp.), and *Braya* Sternb. & Hoppe (22 spp.), as well as 12 monospecific genera.

Because taxa sampling varied in previous studies, the interrelationships among genera of Euclidieae varied a great deal. In order to gain knowledge of phylogenetic relationship of the tribe, we conducted the first comprehensive study that included representatives of all genera.

**Table 1.** Three different taxonomic treatments and current delimitation of Euclidieae. Number of species included in the study and the total species number of the genus based on current delimitation (BrassiBase) are given in parentheses (sampled/total).

**de Candolle (1821)**	**Schulz (1936)**	**Al-Shehbaz (2012)**	**Current delimitation (BrassiBase)**
*Euclidium* W. T. Aiton	*Anastatica* L.	*Atelanthera* Hook. f. & Thomson	*Anzhengxia* Al-Shehbaz & D. A. German (1/1)
*Ochthodium* DC.	*Boreava* Jaub. & Spach	*Braya* Sternb. & Hoppe	*Atelanthera* Hook. f. & Thomson (1/1)
*Pugionium* Gaertn.	*Bunias* L.	*Catenulina* Soják	*Braya* Sternb. & Hoppe (13/22)
	*Euclidium* W. T. Aiton	*Christolea* Cambess.	*Catenulina* Soják (1/ 1)
	*Hymenophysa* C. A. Mey.	*Cryptospora* Kar. & Kir.	*Christolea* Cambess. (1 /2)
	*Lachnoloma* Bunge	*Cymatocarpus* O. E. Schulz	*Cryptospora* Kar. & Kir.(/3)
	*Myagrum* L.	*Dichasianthus* Ovcz. & Yunussov	*Cymatocarpus* O. E. Schulz (1/3)
	*Neslia* Desv.	*Dilophia* Thomson	*Dichasianthus* Ovcz. & Yunussov (1 /1)
	*Ochthodium* DC.	*Euclidium* W. T. Aiton	*Dilophia* Thomson (1/2)
	*Octoceras* Bunge	*Lachnoloma* Bunge	*Euclidium* W. T. Aiton (1/1)
	*Schimpera* Hochst. & Steud. ex Schimper	*Leiospora* (C.A.Mey.) Dvořák	*Lachnoloma* Bunge (1/1)
	*Spirorhynchus* Kar. & Kir.	*Lepidostemon* Hook. f. & Thomson	*Leiospora* (C.A.Mey.) Dvořák (5/8)
	*Tauscheria* Fisch. ex DC.	*Leptaleum* DC.	*Lepidostemon* Hook. f. & Thomson (2/6)
	*Texiera* Jaub. & Spach	*Neotorularia* Hedge & J. Léonard	*Leptaleum* DC. (1/2)
		*Octoceras* Bunge	*Metashangrilaia* Al-Shehbaz & D. A. German (1/1)
		*Pycnoplinthopsis* Jafri	*Neotorularia* Hedge & J. Léonard (4/6)
		*Pycnoplinthus* O. E. Schulz	*Octoceras* Bunge (1/1)
		*Rhammatophyllum* O. E. Schulz	*Pycnoplinthopsis* Jafri (1 /1)
		*Shangrilaia* Al-Shehbaz, J. P. Yue & H. Sun	*Pycnoplinthus* O. E. Schulz (1/1)
		*Sisymbriopsis* Botsch. & Tzvelev	*Rhammatophyllum* O. E. Schulz (5/7)
		*Solms-laubachia* Muschl.	*Rudolf-kamelinia* Al-Shehbaz & D. A. German (1/1)
		*Spryginia* Popov	*Shangrilaia* Al-Shehbaz, J. P. Yue & H. Sun (1/1)
		*Streptoloma* Bunge	*Sisymbriopsis* Botsch. & Tzvelev (3/4)
		*Strigosella* Boiss.	*Solms-laubachia* Muschl. (23/33)
		*Tetracme* Bunge	*Spryginia* Popov (2/7)
			*Streptoloma* Bunge (1/2)
			*Strigosella* Boiss. (3/24)
			*Tetracme* Bunge (3/9)

## Materials and methods

### Plant materials and molecular data

This study comprised 33 genera and 88 species, including 28 genera and 82 species of Euclidieae. Forty-nine ITS sequences of 37 species were newly generated here, and all others were downloaded from GenBank (Table 2 and Appendix 1). Six species of Lineage III (sensu Beilstein et al. 2006), namely *Sterigmostemum sulphureum* (Banks & Sol.) Bornm. and *S. billardieri* (DC.) D. A. German (Anchonieae), *Bunias erucago* L. (Buniadeae), *Clausia aprica* (Stephan ex Willd.) Korn.-Trotzky (Dontostemoneae), and *Hesperis sibirica* L. and *H. isatidea* (Boiss.) D. A. German & Al-Shehbaz (Hesperideae), were used as outgroups.

**Table 2.** List of studied taxa including voucher information and GenBank accession numbers.

Taxon	Geographical origin	Collection number (Herbarium: KUN)	GenBank accession No.
*Anzhengxia yechengnica* (Z. X. An) Al-Shehbaz & D. A. German	Pishan, Xinjiang	YC-XZ111	MH237681
	Yecheng, Xinjiang	YC-XZ115	MH237682
*Braya parvia* (Z. X. An) Al-Shehbaz & D. A. German	Aheqi, Xinjiang	YC-XZ090	MH237683
	Zhada, Xizang	YC-XZ150	MH237684
*Braya rosea* Bunge	Aketao, Xinjiang	YC-XZ105	MH237685
	Kunming, Yunnan	SCSY-042	MH237686
*Braya scharnhorstii* Regel & Schmalh.	Aketao, Xinjiang	YC-XZ101	MH237687
*Christolea crassifolia* Cambess.	Aketao, Xinjiang	YC-XZ103	MH237688
*Dilophia salsa* Thomson	Pishan, Xinjiang	YC-XZ128	MH237689
	Qumalai, Qinghai	ZH645	MH237690
*Euclidium syriacum* (L.) W. T. Aiton	Urumqi, Xinjiang	YC-XZ076	MH237691
*Eurycarpus lanuginosus* (Hook. f. & Thomson) Botsch.	Zhada, Xizang	YC-XZ152	MH237692
			MH237693
			MH237694
*Leiospora eriocalyx* (Regel & Schmalh.) F. Dvořák	Yecheng, Xinjiang	YC-XZ122	MH237695
	Pishan, Xinjiang	YC-XZ125	MH237696
*Leiospora pamirica* (Botsch. & Vved.) Botsch. & Pachom.	Aketao, Xinjiang	YC-XZ102	MH237697
	Aketao, Xinjiang	YC-XZ104	MH237698
*Lepidostemon rosularis* (K. C. Kuan & Z. X. An) Al-Shehbaz	Cuona, Xizang	ZJW3888	MH237699
*Metashangrilaia forrestii* (W. W. Sm.) Al-Shehbaz & D. A. German	Baqing, Xizang	YZC227	MH237700
*Pycnoplinthus uniflora* (Hook. f. & Thomson) O. E. Schulz	Ritu, Xizang	YC-XZ134	MH237701
*Rudolf-kamelinia korolkowii* (Regel & Schmalh.) Al-Shehbaz & D. A. German	Aheqi, Xinjiang	YC-XZ089	MH237702
	Aketao, Xinjiang	YC-XZ107	MH237703
*Shangrilaia nana* Al-Shehbaz, J. P. Yue & H. Sun	Shangrila, Yunnan	CHY008	MH237704
*Sisymbriopsis mollipila* (Maxim.) Botsch.	Yecheng, Xinjiang	YC-XZ119	MH237705
*Sisymbriopsis pamirica* (Y. C. Lan & Z. X. An) Al-Shehbaz, Z. X. An & G. Yang	Aketao, Xinjiang	YC-XZ100	MH237706
*Sisymbriopsis schugnana* Botsch. & Tzvelev	Aketao, Xinjiang	YC-XZ106	MH237707
*Solms-laubachia angustifolia* J. P. Yue, Al-Shehbaz & H. Sun	Daocheng, Sichuan	YZC252	MH237708
*Solms-laubachia baiogoinensis* (K. C. Kuan & Z. X. An) J. P. Yue, Al-Shehbaz & H. Sun	Gongbujiangda, Xizang	YZC195	MH237709
*Solms-laubachia calcicola* J. P. Yue, Al-Shehbaz & H. Sun	Leiwuqi, Xizang	YZC233	MH237710
*Solms-laubachia eurycarpa* (Maxim.) Botsch.	Basu, Xizang	YZC023	MH237711
*Solms-laubachia himalayensis* (Cambess.) J. P. Yue, Al-Shehbaz & H. Sun	Ritu, Xizang	YC-XZ130	MH237712
	Zhada, Xizang	YC-XZ151	MH237713
*Solms-laubachia jafrii* (Al-Shehbaz) J. P. Yue, Al-Shehbaz & H. Sun	Lhasa, Xizang	YZC214	MH237714
	Jilong, Xizang	NZ143	MH237715
*Solms-laubachia kashgarica* (Botsch.) J. P. Yue, Al-Shehbaz & H. Sun	Aheqi, Xinjiang	YC-XZ096	MH237716
*Solms-laubachia lanata* Botsch.	Lhasa, Xizang	YZC215	MH237717
*Solms-laubachia linearlifolia* (W. W. Sm.) O. E. Schulz	Deqin, Yunnan	YZC001	MH237718
*Solms-laubachia linearis* (N. Busch) J. P. Yue, Al-Shehbaz & H. Sun	Pishan, Xinjiang	YC-XZ123	MH237719
*Solms-laubachia mieheorum* (Al-Shehbaz) J. P. Yue, Al-Shehbaz & H. Sun	Angren, Xizang	YC-XZ160	MH237720
*Solms-laubachia platycarpa* (Hook. f. & Thomson) Botsch.	Dangxiong, Xizang	YZC216	MH237721
*Solms-laubachia prolifera* (Maxim.) J. P. Yue, Al-Shehbaz & H. Sun	Mangkang, Xizang	YZC019	MH237722
*Solms-laubachia pulcherrima* Muschl.	Lijiang, Yunnan	ChenHongliang202	MH237723
*Solms-laubachia retropilosa* Botsch.	Xiangcheng, Sichuan	ChenHongliang209	MH237724
*Solms-laubachia villosa* (Maxim.) J. P. Yue, Al-Shehbaz & H. Sun	Yushu, Qinghai	YZC239	MH237725
*Solms-laubachia xerophyte* (W. W. Sm.) H. F .Comber	Shangrila, Yunnan	YZC277	MH237726
*Solms-laubachia zhongdianensis* J. P. Yue, Al-Shehbaz & H. Sun	Shangrila, Yunnan	CHY007	MH237727
*Strigosella africana* (L.) Botsch.	Altay, Xinjiang	YC-XZ031	MH237728
	Yecheng, Xinjiang	YC-XZ117	MH237729

### DNA extraction, PCR amplification, and sequencing

Total genomic DNA was extracted from silica gel-dried leaf materials using the Plant Genomic DNA Kit (Tiangen Biotech, Beijing, China) following the manufacturer’s protocol. The ITS region was amplified with the primers ITS-18F as modified by Mummenhoff et al. (1997) and ITS4 (White et al. 1990). All polymerase chain reactions (PCR) were performed in a 25 μL volume consisting of 1–2μL sample DNA (approx. 1–10 ng), 12.5μL Premix Taq^TM^ (Takara Biomedical Technology, Beijing, China), 1μL of 10 μM stock of each primer, adjusted to 25 μL with ddH_2_O. The PCR protocol of the ITS region involved a hot start with 4 min at 94 °C, and 30–32 cycles of amplification (1 min denaturing at 94 °C, 45–60 s annealing at 52–53 °C, 60–80 s extension at 72 °C), and a final elongation step for 10 min at 72 °C. The sequencing primers are the same as amplified primers.

### Phylogenetic analyses

Original chromatograms were evaluated with Sequencher 4.1.4 (Gene Codes Corporation 2002) for base confirmation and contiguous sequences editing, and sequences were aligned with MAFFT v7.311 (Katoh et al. 2002, Katoh and Standley 2013) and were manually adjusted with MEGA 7.0.14 (Kumar et al. 2016). The aligned sequences were analyzed with maximum parsimony (MP) and Bayesian inference (BI).

Maximum parsimony analysis were performed by PAUP* 4.0a161 (Swofford 2018) with all characters unweighted. Heuristic parsimony searches were conducted with 100 replicates of random addition of sequences to search for multiple islands of most parsimonious trees (Maddison 1991). Bootstrap analyses (BS) (Felsenstein 1985) to assess the relative support for monophyletic groups were calculated from 1000 replicates using a heuristic search with ten random-addition subreplicates, TBR branch swapping and MULPARS in effect. For Bayesian inference analysis, jModeltest v2.1.7 (Darriba et al. 2012) was used to select the best-fitted model of nucleotide substitution based on the Akaike information criterion (AICc), and the SYM+I+G model was selected for the ITS dataset. Bayesian inference based on the Markov chain Monte Carlo methods (Yang and Rannala 1997) was performed using MrBayes v3.2.6 (Ronquist et al. 2012), four simultaneous Monte Carlo Markov chains (MCMCs) were run for five million generations, and one tree sampled every 1000 generations. The first 1250 trees (25% of total trees) were discarded as burn-in. The remaining trees were summarized in a 50% majority-rule consensus tree, and the posterior probabilities (PP) were calculated.

## Results

The aligned ITS dataset comprised 88 species (100 accessions) with 609 characters, of which 256 were variable and 187 (30.7%) were parsimony-informative.

**Figure 1.**
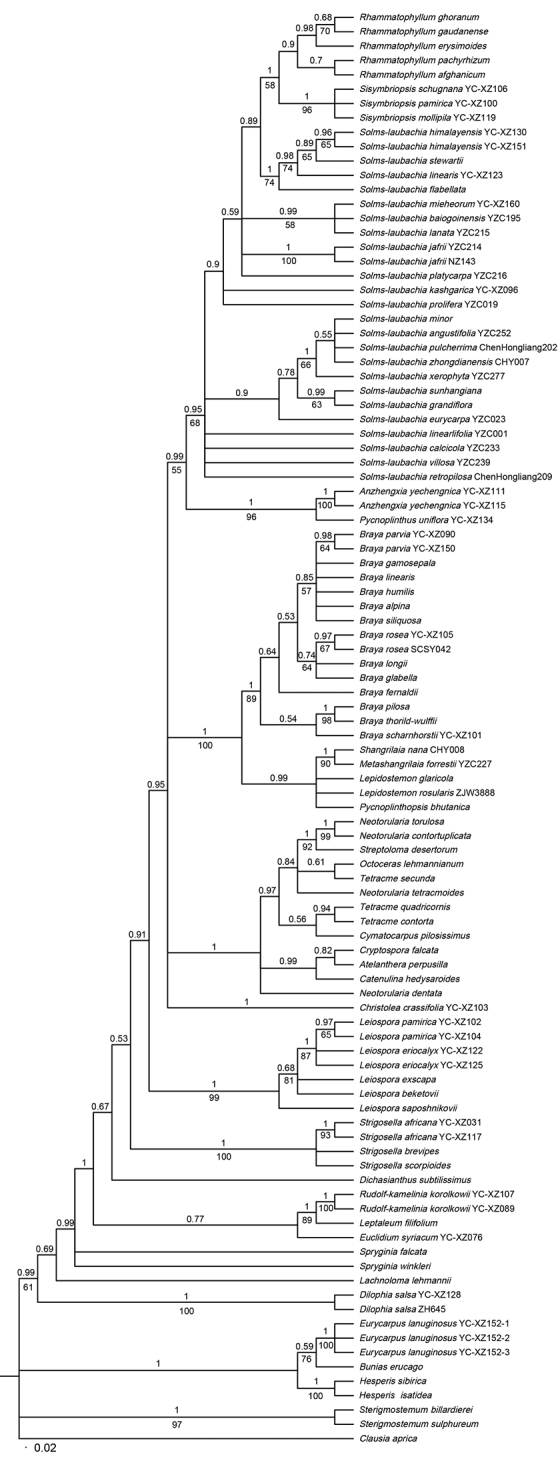
Phylogenetic tree resulting from Bayesian analysis of the ITS sequences of the 88 Brassicaceae species, of which 28 genera and 82 species in Euclidieae. Posterior probabilities are indicated above branches. Bootstrap support values (>50%) are noted below branches.

The resolution of MP analysis was relatively weaker than the outcome of BI analysis. Only the topologies of Bayesian phylogenetic analysis were shown (Figure 1). The result clearly showed that all 28 genera of Euclidieae formed a moderately to strongly supported clade (PP / BS = 0.99 / 61; Figure 1). *Dilophia* Thomson, *Lachnoloma*, and *Spryginia* Popov formed the early branching lineage of the tribe in BI analysis (Figure 1). Five species of *Rhammatophyllum*, three of *Sisymbriopsis*, and 23 of *Solms-laubachia* formed a well-supported subclade within Euclidieae (PP / BS = 0.95 / 68; Figure 1), and then clustered with *Anzhengxia* Al-Shehbaz & D.A.German and *Pycnoplinthus* O.E.Schulz (PP / BS = 0.99 / 55; Figure 1).

All *Braya* species formed a subclade (PP / BS = 1 / 89; Figure 1) sister to *Shangrilaia* Al-Shehbaz, J. P. Yue & H. Sun, *Metashangrilaia* Al-Shehbaz & D. A. German, *Lepidostemon* Hook. f. & Thomson, and *Pycnoplinthopsis* Jafri (Figure 1). Species of *Neotorularia* Hedge & J. Léonard, *Streptoloma* Bunge, *Octoceras* Bunge, *Tetracme* Bunge, *Cymatocarpus* O. E. Schulz, *Cryptospora* Kar. & Kir., *Atelanthera* Hook. f. & Thomson, and *Catenulina* Soják clustered into one clade in BI analysis (PP = 1; Figure 1), whereas both *Neotorularia* and *Tetracme* were found to be polyphyletic. As for the four species of *Neotorularia*, *N. contortuplicata* (Stephan ex Willd.) Hedge & J. Léonard and *N. torulosa* (Desf.) Hedge & J. Léonard formed one clade (PP / BS = 1 / 99; Figure 1), while *N. tetracmoides* (Boiss. & Hausskn.) Hedge & J. Léonard and *N. dentata* (Freyn & Sint.) Hedge & J. Léonard each formed a solitary branch. The three species of *Tetracme* formed two independent subclades in BI analysis, one of which comprised of *T. quadricornis* (Stephan ex Willd.) Bunge and *T. contorta* Boiss. (PP = 0.94; Figure 1), and the other consisted of *T. secunda* Boiss. and *Octoceras lehmannianum* Bunge (PP = 0.61; Figure 1).

In addition to the above clades, species of *Leiospora* (C. A. Mey.) Dvořák and *Strigosella* formed two well supported clades, suggesting that both are monophyletic. However, *Solms-laubachia lanuginosa* (Hook. f. & Thomson) D. A. German & Al-Shehbaz (formerly *Eurycarpus lanuginosus* (Hook. f. & Thomson) Botsch.) did not fall within the *Solms-laubachia*-*Rhammatophyllum*-*Sisymbriopsis* clade. Instead, three accessions of this species formed a clade with outgroup taxa *Bunias erucago* (PP / BS = 0.59 / 76; Figure 1), indicating that *S. lanuginosa* is neither a member of genus *Solms-laubachia* nor of the tribe Euclidieae.

## Discussion

Our results suggested that *Solms-laubachia* s.l. is not monophyletic, within which both *Rhammatophyllum* and *Sisymbriopsis* are embedded. Besides, *S. lanuginosa* fell outside of the clade. The closeness of *Solms-laubachia*, *Rhammatophyllum*, and *Sisymbriopsis* was revealed in previous studies (e.g., Belstein et al. 2006, 2008, Warwick et al. 2007, German et al. 2009). However, these studies only sampled one or two representative species of each genus and therefore did not reach a convincible conclusion on their generic status. By contrast, this study sampled 23 of 33 species of *Solms-laubachia*, five of seven of *Rhammatophyllum*, and three of four of *Sisymbriopsis*, representing thus far the most complete taxa sampling on these three genera.

*Solms-laubachia* had recently been subjected to a series of studies, including taxonomy (Lan and Zhou 1981, Al-Shehbaz and Yang 2000), cytology (Yue et al. 2003, 2004), molecular phylogeny (Yue et al. 2006, 2008), and biogeography (Yue et al. 2009). As traditionally circumscribed, this genus contained nine to thirteen species distributed from Southwest China to East-Himalayan. However, molecular phylogenetic studies demonstrated that *Desideria* Pamp. and *Phaeonychium* O. E. Schulz should be included in it, and that led to greatly expanding of the morphological and geographic boundaries of *Solms-laubachia*. For example, previously delimited *Solms-laubachia* species have entire, pinnately veined leaves and latiseptate fruit, whereas the expanded *Solms-laubachia* also has palmately veined leaves, and terete fruit. The geographic distribution of *Solms-laubachia* s.l. is also expanded westward into Central Asia.

*Rhammatophyllum* consists of shrubs or subshrubs with soft malpighiaceous, submalpighiaceous, or rarely subdendritic trichomes, filiform to linear or lanceolate, entire cauline leaves, and dehiscent fruit with torulose valves. Its seven species are distributed from Turkmenistan and W Kazakhstan into W Mongolia (Botschantzev 1987, Al-Shehbaz and Appel 2002, Kamelin 2002, German et al. 2006, Moazzeni et al. 2014). By contrast, *Sisymbriopsis* includes annual, biennial or perennial herbs primarily with stalked and 1- or 2-forked to dendritic trichomes, pinnately lobed to coarsely dentate or rarely subentire basal and cauline leaves, and linear, flattened and latiseptate fruit with torulose valves and complete septum. Its four species are distributed in Afghanistan, China (Qinghai, Xinjiang, and Xizang), Kyrgyzstan, and Tajikistan (Al-Shehbaz et al. 1999, Al-Shehbaz and German 2016).

Although our results suggest combining *Solms-laubachia*, *Rhammatophyllum*, and *Sisymbriopsis* into one monophyletic genus, merging these three genera into one will make it vastly heterogeneous morphologically (Table 3).The combined genus would be highly variable by encompassing nearly all habit types in the family, nearly all petals colors, and almost all inflorescence types, and would be almost impossible to delimit morphologically. Alternatively, one could keep both *Rhammatophyllum* and *Sisymbriopsis* as separate monophyletic genera (Figure 1), and split *Solms-laubachia* s.l. into several well-delimited smaller genera depending on how different the species cluster together. Because our phylogenetic analyses was based on single ITS sequence fragments, infra-generic relationships can be satisfactorily resolved only by further studies dealing with cpDNA and other single-copy nuclear markers.

**Table 3.** Comparsions on morphological characters of *Solms-laubachia*, *Sisymbriopsis*, *Rhammatophyllum*, and *Eurycarpus*.

	*Sisymbriopsis*	*Rhammatophyllum*	*Solms-laubachia*	*Eurycarpus*
Habit	annual, biennial, or perennial herbs	shrubs or subshrubs	perennial herbs	perennial herbs
Trichomes	simple and/or stalked forked or dendritic	softly malpighiaceous, submalpighiaceou, rarely subdendritic	absent or simple, rarely short-stalked, 2-rayed	simple mixed with stalked 1- to 3-forked ones
Basal leaves	rosulate or not	not rosulate	rosulate	rosulate
Leaf margin	dentate, rarely subentire	entire	entire or 3- to 9(to 11)-toothed	entire
Leaf venation	pinnate	pinnate	pinnate or palmate	pinnate
Cauline leaves	present	present	present or absent	absent
Flower	in racemes, ebracteate or bracteate corymbose	in racemes, ebracteate corymbose	solitary or in racemes, ebracteate or bracteate corymbose	in racemes, ebracteate corymbose
Sepals	equal, nonsaccate	subequal, nonsaccate	equal, nonsaccate	equal, nonsaccate
Petal colour	white or lavender	yellow, creamy white, or rarely purple	purple, blue, pink, or rarely white	purple
Anther apex	obtuse or apiculate	apiculate	obtuse	obtuse
Anther shape	ovate or oblong	oblong	oblong-linear to ovate	oblong
Median nectaries	present	absent or present	absent or present	present
Fruit shape	dehiscent silicles, linear, flattened and latiseptate	dehiscent siliques, linear, latiseptate	dehiscent silique or silicle, linear, oblong, ovate, lanceolate, or ellipsoid, latiseptate or terete	dehiscent silicles, oblong, elliptic, ovate-oblong, or ovate-lanceolate, strongly latiseptate
Fruit valve	valves papery, prominently veined, glabrous or pubescent, torulose	valves papery, prominently veined, pubescent, torulose	valves papery, reticulate veined, glabrous or pubescent, smooth or torulose	valves obscurely veined, glabrous, smooth
Septum	complete	complete	complete or rarely perforated or reduced to a rim	complete or reduced to a rim
Style	obsolete	obsolete or distinct	obsolete or distinct	obsolete
Stigma	capitate, entire or 2-lobed, lobes not decurrent	capitate, entire or 2-lobed, lobes not decurrent	capitate, entire or 2-lobed, lobes not decurrent	capitate, entire
Seed	uniseriate, wingless or rarely distally with a small wing	uniseriate, winged, margined, or wingless	uniseriate or biseriate, wingless, seed coat reticulate, rugose, or papillate, not mucilaginous when wetted	biseriate, wingless, seed coat minutely reticulate, not mucilaginous when wetted
Cotyledons	obliquely accumbent	accumbent or rarely incumbent	accumbent	incumbent or accumbent

As for the outlier *Solms-laubachia lanuginosa*, its three accessions formed a clade clustered with *Bunias erucago* (Buniadeae), *Hesperis sibirica*, and *H. isatidea* (Hesperideae). Because it fell out of *Solms-laubachia* and the remainder of Euclidieae, we suggest restoring its previous status in the genus *Eurycarpus* Botsch. The incongruence between taxonomic treatments based strictly on morphology call for the need to draw generic limits and relationships after conducting adequate molecular phylogenetic analyses. Identifying the tribal position of *Eurycarpus* is beyond the scope of this paper, and it will be conducted in the near future with nuclear and chloroplast sequences data.

The monospecific genus *Metashangrilaia* was established based on *M. forrestii* (W.W.Sm.) Al-Shehbaz & D.A.German, a species used to be put in *Braya*. Previous molecular analyses revealed that it had very distinct ITS sequences and formed a well-supported clade sister to the rest of *Braya* (Warwick et al. 2004). Besides, it showed great morphological divergences from other *Braya* species (Al-Shehbaz et al. 2004, Al-Shehbaz and German 2016). All these led Al-Shehbaz and German (2016) to separate it from *Braya* and accommodate it in the newly established *Metashangrilaia*. This study provides further evidence on a strong sister taxon relationship between *Metashangrilaia* and *Shangrilaia* (Figure 1), supporting the decision by Al-Shehbaz and German (2016).

Our results also suggest that *Neotorularia*, *Tetracme*, and *Lepidostemon* are not monophyletic. Of the four species sampled from *Neotorularia*, the generic type *N. torulosa* clustered with *N. contortuplicata*, and they were sister to *Streptoloma desertorum* Bunge, while *N. tetracmoides* and *N. dentata* each formed an independent clade (Figure 1). The three sampled *Tetracme* formed two separate clades, one of which was *T. contorta* and *T. quadricornis*, whereas the other was *T. secunda* and *Octoceras lehmannianum* (Figure 1). The non-monophyly of both genera is congruent with previous studies (Warwick et al. 2004, 2007).

Finally, *Lepidostemon* used to be a monospecific genus, the type species is *L. pedunculosus* Hook. f. & Thomson. It was expanded by Al-Shehbaz (2000, 2002), to include six species endemic to the Mid-western Himalaya (Al-Shehbaz 2015). The ITS sequence of *L. glaricola* (H. Hara) Al-Shehbaz (Couvreur et al. 2010) did not fall with our newly sequenced *L. rosularis* (K. C. Kuan & Z. X. An) Al-Shehbaz in one clade. However, due to limited data and low solution of ITS sequences, further studies with extensive sampling and more molecular markers are needed to clarify the taxonomic circumscription of the non-monophyletic genera – *Neotorularia*, *Tetracme*, and *Lepidostemon*.

## Appendix 1

Taxa and accession numbers downloaded from GenBank for the ITS sequences used in the study.

**Outgroups**: Tribe Anchonieae: *Sterigmostemum billardierei* (DC.) D. A. German (DQ357513), *S. sulphureum* (Banks & Sol.) Bornm. (KJ663764). Tribe Buniadeae: *Bunias erucago* L. (GQ497885). Tribe Dontostemoneae: *Clausia aprica* (Stephan ex Willd.) Korn.-Trotzky (LK021257). Tribe Hesperideae: *Hesperis isatidea* (Boiss.) D. A. German & Al-Shehbaz (GQ497882); *Hesperis sibirica* L. (DQ357549). **Ingroups**: Tribe Euclidieae: *Atelanthera perpusilla* Hook. f. & Thomson (FM164518, FM164519); *Braya alpina* Sternb. & Hoppe (AY353096), *B. fernaldii* Abbe (AY353152), *B. gamosepala* (Hedge) Al-Shehbaz & S. I. Warwick (AF137565), *B. glabella* Richardson (AF137578), *B. humilis* (C. A. Mey.) B. L. Rob. (AY237325), *B. linearis* Rouy (AY353102), *B. longii* Fernald (AY237310), *B. pilosa* Hook. (KT727927), *B. siliquosa* Bunge (AY353105), *B. thorild-wulffii* Ostenf. (AY353098); *Catenulina hedysaroides* (Botsch.) Soják (GQ424607); *Cryptospora falcata* Kar. & Kir. (DQ357532); *Cymatocarpus pilosissimus* (Trautv.) O. E. Schulz (GQ497858); *Dichasianthus subtilissimus* (Popov) Ovcz. & Junussov (AY353169); *Lachnoloma lehmannii* Bunge (GQ497889); *Leiospora beketovii* (Krasn.) D.A. German & Al-Shehbaz (FN821579); *L. exscapa* (Ledeb.) F. Dvořák (FN821615), *L. saposhnikovii* (A.N. Vassiljeva) D.A. German & Al-Shehbaz (FN821554); *Lepidostemon glaricola* (H.Hara) Al-Shehbaz (GQ424542); *Leptaleum filifolium* (Willd.) DC. (KJ623485); *Neotorularia contortuplicata* (Stephan ex Willd.) Hedge & J. Léonard (AY353165), *N. dentata* (Freyn & Sint.) Hedge & J. Léonard (AY353160), *N. tetracmoides* (Boiss. & Hausskn.) Hedge & J. Léonard (AY353162), *N. torulosa* (Desf.) Hedge & J. Léonard (AY353167); *Octoceras lehmannianum* Bunge (GQ424609); *Pycnoplinthopsis bhutanica* (H. Hara) Jafri (GQ497878); *Rhammatophyllum afghanicum* (Rech. f.) Al-Shehbaz & O. Appel (DQ357583), *R. erysimoides* (Kar. & Kir.) Al-Shehbaz & O. Appel (DQ357587), *R. gaudanense* (Litv.) Al-Shehbaz & O. Appel (DQ357585), *R. ghoranum* (Rech. f.) Al-Shehbaz & O. Appel (DQ357586), *R. pachyrhizum* (Kar. & Kir.) O. E. Schulz (DQ357588); *Solms-laubachia flabellata* (Regel) J. P. Yue, Al-Shehbaz & H. Sun (GQ497886), *S. grandiflora* J.P. Yue, Al-Shehbaz & H. Sun (DQ523419), *S. minor* Hand.-Mazz. (DQ523418), *S. stewartii* (T.Anderson) J. P. Yue, Al-Shehbaz & H. Sun (FN821609), *S. sunhangiana* J.P. Yue & Al-Shehbaz (EU186027); *Spryginia falcata* Botsch (FN821518), *S. winkleri* (Regel) Popov (GQ424563); *Streptoloma desertorum* Bunge (FM164618, FM164619); *Strigosella brevipes* (Bunge) Botsch. (DQ357558), *S. scorpioides* (Bunge) Botsch. (KJ623536); *Tetracme contorta* Boiss. (DQ357600), *T. quadricornis* (Stephan ex Willd.) Bunge (DQ357602), *T. secunda* Boiss. (DQ357604).
